# Numerical Investigation on Electrothermal Performance of AlGaN/GaN HEMTs with Nanocrystalline Diamond/SiNx Trench Dual-Passivation Layers

**DOI:** 10.3390/nano15080574

**Published:** 2025-04-10

**Authors:** Peiran Wang, Chenkai Deng, Chuying Tang, Xinyi Tang, Wenchuan Tao, Ziyang Wang, Nick Tao, Qi Wang, Qing Wang, Hongyu Yu

**Affiliations:** 1 School of Microelectronics, Southern University of Science and Technology, Shenzhen 518055, China; 12333281@mail.sustech.edu.cn (P.W.); 12149033@mail.sustech.edu.cn (C.D.); 12049024@mail.sustech.edu.cn (C.T.); 12232507@mail.sustech.edu.cn (X.T.); 12333343@mail.sustech.edu.cn (W.T.); 12333347@mail.sustech.edu.cn (Z.W.); 2 School of Electronic Information and Engineering, Harbin Institute of Technology, Harbin 150001, China; 3 Maxscend Microelectronics Co., Ltd., Wuxi 214072, China; nick.tao@maxscend.com; 4 Dongguan Institute of Opto-Electronics, Peking University, Dongguan 523820, China; wangq@pku-ioe.cn; 5 Engineering Research Center of Integrated Circuits for Next-Generation Communications, Ministry of Education, Southern University of Science and Technology, Shenzhen 518055, China; 6 School of Integrated Circuit, Shenzhen Polytechnic University, Shenzhen 518055, China

**Keywords:** AlGaN/GaN HEMTs, numerical simulations, nanocrystalline diamond, trench dual-passivated structure, self-heating effects

## Abstract

In this work, AlGaN/GaN high-electron-mobility transistors (HEMTs) with a nanocrystalline diamond (NCD)/SiN_x_ trench dual-passivated (TDP) structure were promoted, which demonstrated superior performance with a higher saturation output current (*I*_dss_) of 1.266 A/mm, a higher maximum transconductance (*G*_mmax_) of 0.329 S/mm, and a lower resistance (*R*_on_) of 2.64 Ω·mm. Thermal simulations revealed a peak junction temperature of 386.36 K for TDP devices under *V*_ds_/*V*_gs_ = 30 V/0 V, representing 13.7% and 4.5% reductions versus SiN_x_ single-passivated (SP, 447.59 K) and dual-passivated (DP, 404.58 K) devices, respectively. The results suggested that compared to conventional SP and DP devices, TDP devices can effectively suppress the self-heating effect, thereby improving output characteristics while maintaining superior RF small-signal characteristics. Moreover, the results of numerical simulations indicated that the enhanced electrothermal performance of TDP devices was predominantly attributed to the mitigation of temperature-induced degradation in electron mobility and drift velocity, thereby preserving their high power and high frequency capabilities. These results highlighted the significant potential of TDP devices to improve the performance of GaN HEMTs in high-power and high-frequency applications.

## 1. Introduction

AlGaN/GaN high-electron-mobility transistors (HEMTs) are excellent candidates for high-frequency, high-voltage and high-power applications due to their superior semiconductor material properties [1]. However, in microwave high-power applications, AlGaN/GaN HEMTs generate a substantial amount of heat alongside a high-power output, known as the self-heating effect [2]. If the heat generated from self-heating does not rapidly and efficiently dissipate, it could substantially raise the device’s peak temperature and consequently lead to the degradation of DC characteristics, output power and efficiency [3].

Several thermal management techniques have been implemented in AlGaN/GaN HEMTs. The most straightforward approach involves using materials with high thermal conductivity adjacent to the chip’s hotspot to minimize temperature increase [4,5,6]. These heat-spreading methods included the growth of AlGaN/GaN on single-crystal or CVD diamond substrates [7,8,9]. However, the use of diamond substrates on the bottom side has encountered limitations related to the limited size of the substrate. Recently, a method involving the integration of heat-spreading films into the top-side structure has been proposed [10,11,12,13,14,15,16], in which high-thermal-conductivity (up to 1300 W/m-K) nanocrystalline diamond (NCD) worked as an effective heat-spreading material. In previous studies, NCD top-side thermal spreading layers were mainly utilized in GaN power devices [17,18,19]. Due to the complicated thick NCD layer etching process following the submicron critical dimension gate process, it is difficult to apply these layers to RF devices [20]. Ulm University achieved impressive small-signal performance (*f*_T_ = 4.2 GHz, *f*_max_ = 5 GHz) using 0.25 μm gate length InAlN/GaN HEMTs encapsulated in NCD [21]. A barrier protection layer, such as SiN_x_ no less than 20 nm, is usually involved to prevent the impaction of plasma damage generated by the NCD deposition process on the AlGaN/GaN heterojunction [22]. Hence, conventional NCD thermal spreading layers function as NCD/SiN_x_ dual-passivation layers. Nevertheless, the structure engineering and performance impaction of the NCD/SiN_x_ heat dissipation layer has not been investigated or reported yet.

In this paper, an electrothermal numerical investigation was performed to systematically compare the thermal, DC and RF performance of conventional SiN_x_ single-passivated (SP), nanocrystalline diamond (NCD)/SiN_x_ dual-passivated (DP) and NCD/SiN_x_ trench dual-passivated (TDP) AlGaN/GaN HEMT devices. TDP devices demonstrated the lowest peak junction temperature among the DP and SP devices at the same bias condition, which shows its novel structure that can further suppress self-heating effects based on a top-side heat dissipation technology roadmap, which matches well with the results of the simulation of DC, RF small-signal and parasitic capacitance characteristics. Combined with static electron mobility and electron drift velocity distribution analyzation, the impaction of suppressed self-heating effects was revealed.

## 2. Device Structure Design and Simulation Setup

**Figure 1:**
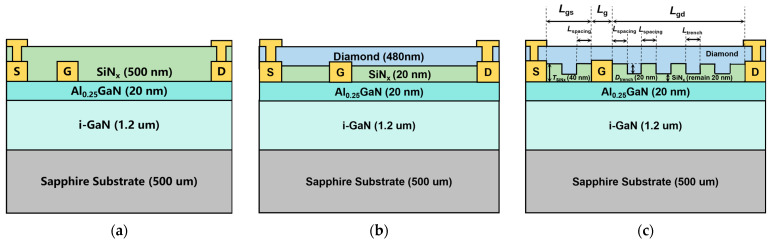
Cross-sectional schematics of (**a**) SP, (**b**) DP and (**c**) TDP AlGaN/GaN HEMTs.

The schematics of SP, DP and TDP devices implemented for TCAD simulations are shown in Figure 1a–c. The AlGaN/GaN HEMTs consist of a 20 nm Al_0.2_Ga_0.8_N barrier layer with a 1.2 um i-GaN channel layer and a sapphire substrate. For SP, DP and TDP devices, the thickness of the passivation layer is 500 nm SiN_x_, 480/20 nm NCD/SiN_x_ and 460/40 nm NCD/SiN_x_ to control the total passivation layers’ thickness equally. For TDP, the depth, length and spacing of the trench are 20 nm, 100 nm and 100 nm, respectively. Therefore, the thickness of SiN_x_ at the trench bottom is 20 nm, which can protect the barrier layer effectively during the NCD deposition process. The simulated device features a gate length (*L*_g_) of 100 nm, a gate-source distance (*L*_gs_) of 1.6 μm and a gate-drain distance (*L*_gd_) of 2.4 μm.

In this simulation work, the semiconductor parameters of the GaN and AlGaN material were set up as shown in Table 1 according to the calibration results from the works of Karmalkar et al. [23]. The interface trap, located at 0.6 eV below the conduction band edge, was used. The areal density of the interface traps used in this work was 2 × 10^12^/cm^2^. The acceptor trap concentration in the buffer layer was 1 × 10^18^/cm^2^ at a trap energy location of 0.8 eV below the conduction band edge. The capture cross-sections for electrons and holes were 1 × 10^−15^/cm^2^ with a degeneracy factor of one. The models incorporated for the simulations were as follows: FERMI for the fermi statistic model; SRH for the carrier generation and recombination model; FLDMOB and ALBRCT to account for the mobility and saturation velocity effects; GANSAT for the nitride-specific mobility model; the IMPACT SELB impact ionization model for activating avalanche breakdown simulations; the trap model to configure the trap effects; and CALC.STRAIN to calculate the strain and the POLARIZATION model invoked for epitaxial strain due to the lattice mismatch and spontaneous polarization.

**Figure 2:**
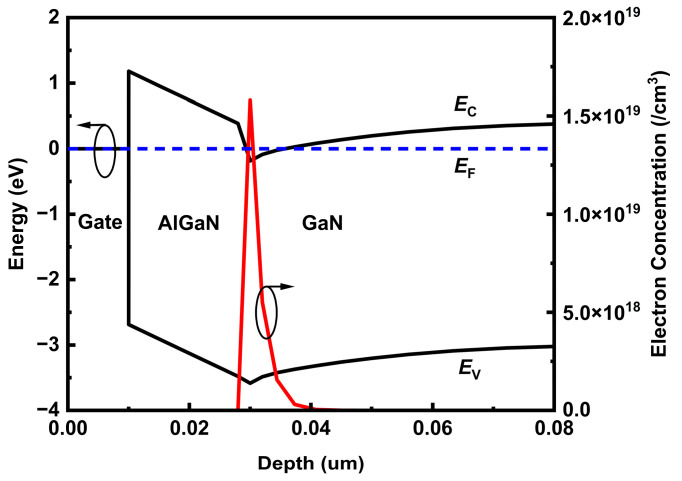
Band diagram and electron concentration distribution extracted from the gate to AlGaN and stopped at GaN layers.

On account of the gate, AlGaN and GaN epilayers of SP, DP and TDP devices are the same simulated configurations; the energy band and electron concentration in the AlGaN/GaN heterojunction are conformed apparently. Figure 2 shows the conduction band energy and valence band energy vertically extracted from the gate to AlGaN and stopped in the GaN layer. The classic band diagram and electron distribution are displayed to verify the static electric characteristics in the GaN HEMT device model functioned reasonably. The peak electron concentrations at the interface of AlGaN and GaN are 1.58 × 10^19^/cm^3^.

Both NCD (10 W/cm-K) and SiN_x_ (0.2 W/cm-K) are considered for device passivation layers. The thermal conductivity of AlGaN, GaN and sapphire substrate was set as 0.13, 1.8 and 0.35 W/cm-K [24]. The bottom of the structure was set to a fixed temperature of 300 K for SP, DP and TDP device simulations.

## 3. Results and Discussion

### 3.1. Junction Temperature Distributtion

**Figure 3:**
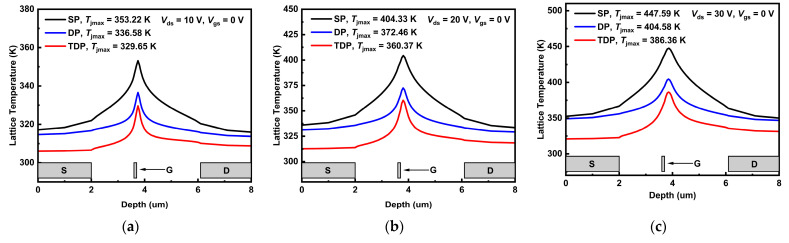

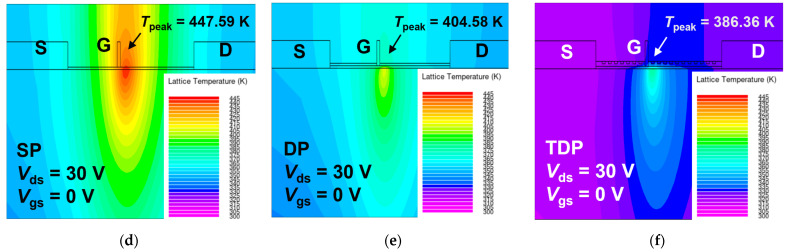
Junction temperature distribution of the SP, DP and TDP AlGaN/GaN HEMTs biased at (**a**) *V*_ds_/*V*_gs_ = 10 V/0 V, (**b**) *V*_ds_/*V*_gs_ = 20 V/0 V and (**c**) *V*_ds_/*V*_gs_ = 30 V/0 V. For *V*_ds_/*V*_gs_ = 30 V/0 V, cross-sectional lattice temperature profiles of the (**d**) SP, (**e**) DP, and (**f**) TDP devices for *V*_ds_/*V*_gs_ = 30 V/0 V are presented.

The temperature distributions along the AlGaN/GaN heterojunction in SP, DP and TDP devices are shown in Figure 3a–c. It can be observed that the junction temperature of SP, DP and TDP devices increases as the bias increase from *V*_ds_/*V*_gs_ = 10 V/0 V to *V*_ds_/*V*_gs_ = 30 V/0 V. Furthermore, TDP devices exhibit the lowest average temperature biased at *V*_gs_ = 0 V and *V*_ds_ = 10 V to 30 V compared to SP and DP devices. In particular, the peak junction temperature of TDP devices is 386.36 K biased at *V*_ds_/*V*_gs_ = 30 V/0 V, which is much lower than DP (404.58 K) and SP (447.59 K) devices. In Figure 3d,e, the cross-sectional lattice temperature profiles of SP, DP and TDP devices biased at *V*_ds_/*V*_gs_ = 30 V/0 V are presented with a normalized temperature scale bar. The trench structure combined the NCD and SiN_x_ layers assist the horizontal heat dissipation so that the heat source region expands toward the gate-to-source access region. Therefore, TDP devices achieve better thermal management capability compared to conventional DP and SP devices.

According to the working principle of the AlGaN/GaN HEMT device, it is known that a higher current and power result in increased device temperature, which in turn leads to a greater probability of defects and easier degradation. As reported in previous research [25], every 25-kelvin degree junction temperature increases from 398 K to 448 K and the value of the mean time to failure of the GaN HEMTs decreases by an order of magnitude. Therefore, the TDP structure has been shown to significantly enhance the devices’ performance, along with improvements in reliability and lifetime.

### 3.2. DC Characteristics and RF Small-Signal Characteristics

**Figure 4:**
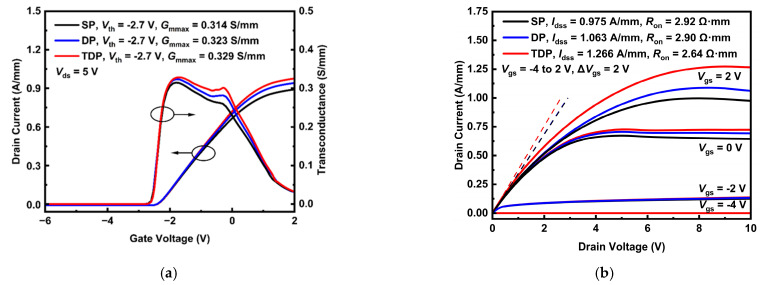
(**a**) Transfer characteristics and (**b**) output characteristics of SP, DP and TDP AlGaN/GaN HEMTs.

Figure 4a shows the typical DC transfer characteristics and transconductance curves of SP, DP and TDP devices. The results reveal that the threshold voltage of the devices is not impacted significantly by the SiN_x_ and NCD structure. In addition, transconductance (*G*_m_) of GaN HEMTs plays a critical role in device linearity and noise, which are important parameters in RF devices. According to the results, the maximum transconductance (*G*_mmax_) is 0.329 S/mm for TDP devices, which is slightly higher than that of 0.314 S/mm and 0.323 S/mm for SP and DP devices.

The output characteristics of SP, DP and TDP devices are shown in Figure 4b. The output characteristics of SP and DP devices are degraded due to the severe self-heating effects. The self-heating phenomena occur in the saturation region of output characteristic and are improved significantly in the AlGaN/GaN HEMTs with TDP structure: as the *V*_gs_ increases, the improvement increases. TDP devices demonstrate the highest saturation drain current (*I*_dss_) of 1.266 A/mm and lowest conduction resistance (*R*_on_) of 2.64 Ω·mm compared to *I*_dss_ = 1.063 A/mm and 0.975 A/mm, *R*_on_ = 2.90 Ω·mm and 2.92 Ω·mm for DP and SP devices. These results indicate a 19.1% and 29.8% *I*_dss_ improvement of TDP devices compared to DP and SP devices. The improvement in *I*_dss_ for the TDP devices is attributed to the degradation of temperature-dependent mobility and 2DEG electron drift velocity.

**Figure 5:**
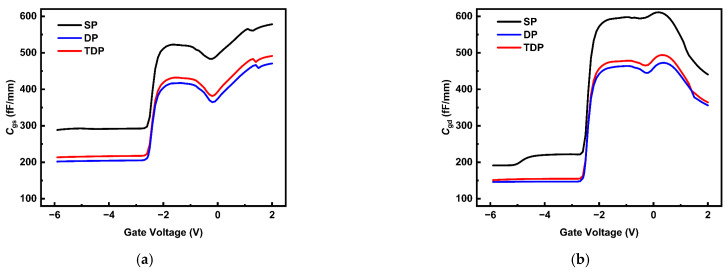
(**a**) *C*_gs_ (**b**) *C*_gd_ related to gate voltage for SP, DP, TDP AlGaN/GaN HEMT devices.

The parasitic capacitances *C*_gs_ and *C*_gd_ as functions of *V*_gs_ (ranging from −6 V to 2 V) are shown in Figure 5. The higher parasitic capacitance observed in SP devices primarily arises from the larger relative permittivity of SiN_x_ (~7 [26]) compared to NCD (~5.6 [27]). Replacing portions of SiN_x_ with NCD in DP and TDP devices reduces the effective permittivity, thereby lowering their parasitic capacitances to different extents. Consequently, the SP device exhibits the highest effective permittivity and parasitic capacitance among the three structures. Additionally, the trench design in TDP devices results in a slightly larger volume of SiN_x_ than in DP devices, leading to a marginally higher effective permittivity and parasitic capacitance in TDP compared to DP. This trade-off between parasitic capacitance and maximum transconductance explains why the TDP device’s peak *f_T_* and *f*_max_ values are slightly lower than those of the DP device. Nevertheless, the TDP structure maintains superior linearity. Importantly, the NCD/SiN_x_ passivation significantly suppresses parasitic capacitance overall, directly influencing the frequency-dependent performance trends discussed in subsequent sections.

**Figure 6:**
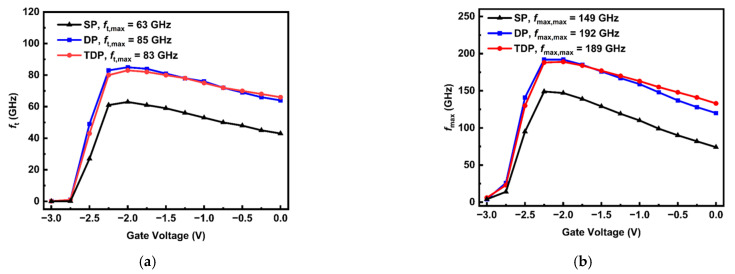
(**a**) *f*_t_ and (**b**) *f*_max_ related to gate voltage for SP, DP, TDP AlGaN/GaN HEMT devices.

The typical small-signal characteristics *f*_t_ and *f*_max_ related to the gate voltage for the SP, DP and TDP devices are presented in Figure 6. The cut-off frequency (*f*_t_) value is extracted by extrapolating the |*H*_21_|^2^ parameter, where the slope is −20 dB/dec and the gain reaches 0 dB. The maximum oscillation frequency *f*_max_ value is extracted from the unilateral power gain, where the slope is −20 dB/dec and the gain reaches 0 dB. Compared to the SP device, the DP and TDP devices exhibit higher *f*_t_ and *f*_max_ as *V*_gs_ varying from −3 V to 0 V.

The maximum *f*_t_ and *f*_max_ for the TDP device are slightly lower than those of the DP device. However, the TDP device demonstrates higher linearity due to its transconductance distribution being flatter than the DP device. Furthermore, the passivation layer materials function to introduce parasitic capacitances among all the electrodes, which in turn limit the device performance, resulting in lower *f*_t_ and *f*_max_ max values. This effect is particularly significant in mm-wave devices, where the *L*_g_ and *L*_sd_ are shorter, *f*_t_ and *f*_max_ are considerably sensitive to the parasitic capacitances (i.e., *C*_gs_ and *C*_gd_). The equations for *f*_t_ and *f*_max_ are Equations (1) and (2) [28]:

(1) $$f_{t}=\frac{g_{m}}{2\pi \left(C_{gs}+C_{gd}\right)}\;$$

(2) $$f_{max}=\frac{f_{t}}{2\sqrt{\frac{R_{i}+R_{s}+R_{g}}{R_{ds}+2\pi f_{t}R_{g}C_{gd}}}}\;$$

### 3.3. Electron Mobility and Electron Drift Velocity Distributtion

**Figure 7:**
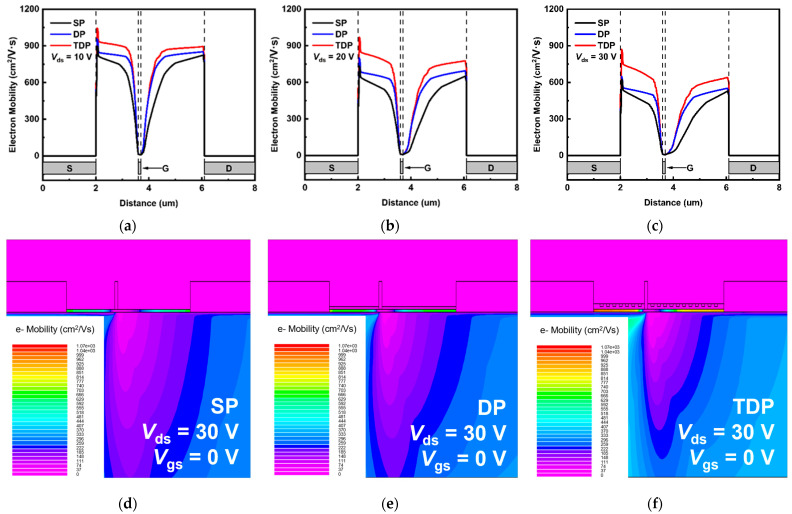
Electron mobility of junctional region in the SP, DP and TDP AlGaN/GaN HEMTs biased at (**a**) *V*_ds_/*V*_gs_ = 10 V/0 V, (**b**) *V*_ds_/*V*_gs_ = 20 V/0 V and (**c**) *V*_ds_/*V*_gs_ = 30 V/0 V. For *V*_ds_ = 30 V, cross-sectional electron mobility distributions of the (**d**) SP, (**e**) DP, and (**f**) TDP AlGaN/GaN HEMTs are presented.

As a parameter strongly dependent on both temperature and electric field, electron mobility decreases with increasing temperature and electric field due to the increased optical phonon scattering [29]. In Figure 7, the SP, DP and TDP devices biased at *V*_ds_/*V*_gs_ = 10 V/0 V, 20 V/0 V, 30 V/0 V are shown. It is observed that the channel under the gate electrode exhibits the lowest electron mobility compared to the channel between the gate and the source/drain. This is because the electric field and lattice temperature peaks are located at the drain-side channel under the gate.

In Figure 7a–c, and as shown in Figure 3a–c, it is evident that under the same *V*_ds_ bias condition, the average channel temperature of the TDP device is lower compared to DP and SP devices. Therefore, according to the compensation mobility model, the low-field mobility of the TDP device is higher than that of the DP and SP devices. The low-field mobility model used in this simulation is as follows [30]:

(3) $$\frac{1}{\mu (N,T_{L})}=a\left(\frac{N_{I}}{10^{17}cm^{-3}}\right)\ln\left(1+\beta_{CW}^{2}\right){\left(\frac{T}{300\;K}\right)}^{-1.5}+b{\left(\frac{T}{300\;K}\right)}^{1.5}+\frac{c}{\exp\left(\frac{\Theta }{T}\right)-1},\;$$

where *µ*(*N*,*T_L_*) is the mobility as a function of total doping concentration (*N*) and lattice temperature (*T*_L_). Though AlGaN/GaN layers in SP, DP and TDP devices are defined as un-intended doping, GaN materials inherently exhibit an unintentional background doping concentration (1 × 10^16^/cm^3^) due to residual oxygen/silicon impurities during epitaxial growth [31]. Therefore, *N* equals to 1 × 10^16^/cm^3^ as the background doping concentration. Additionally, $N_{I}=\left(1+k_{c}\right)N_{D},\;\beta_{CW}^{2}=3.00{\left(\frac{T}{300\;K}\right)}^{2}{\left(\frac{N_{I}}{10^{17}cm^{-3}}\right)}^{-\frac{2}{3}},\;\Theta =\frac{\hbar \omega_{LO}}{k_{B}}=1065\;K.\;$For parameters $a=2.61\times 10^{-4}\;V\;scm^{-2},\;b=2.90\times 10^{-4\;}Vscm^{-2},\;c=1.70\times 10^{-2}\;Vscm^{-2}.\;$Therefore, as the lattice temperature increases, the electron mobility along the channel will degrade. Moreover, as the *V*_ds_ increases, electrons within the AlGaN/GaN HEMT devices channel acquire energy through the electric field generated by the bias voltage. Consequently, during their drift or diffusion motion, these electrons collide with the lattice, transferring energy to the lattice in the form of phonons. This process subsequently leads to an increase in the lattice temperature and a decrease in electron mobility.

Figure 7d–f illustrate the electron mobility distribution for SP, DP, and TDP devices under the same bias voltage conditions (*V*_ds_/*V*_gs_ = 30 V/0 V). With the high thermal conductivity layer NCD introduced, the total thermal conductivity of the passivation layer decreased efficiently. In addition, the trench structure coupled with NCD and SiN_x_ layers can increase the contact area of the thermal interface so that the effective thermal resistance at the NCD/SiN_x_ interface is reduced. As a result, it is evident that, due to the excellent heat dissipation effect of the TDP structure, the electron mobility in the two-dimensional plane of the TDP device is significantly higher than that of the DP and SP devices.

**Figure 8:**
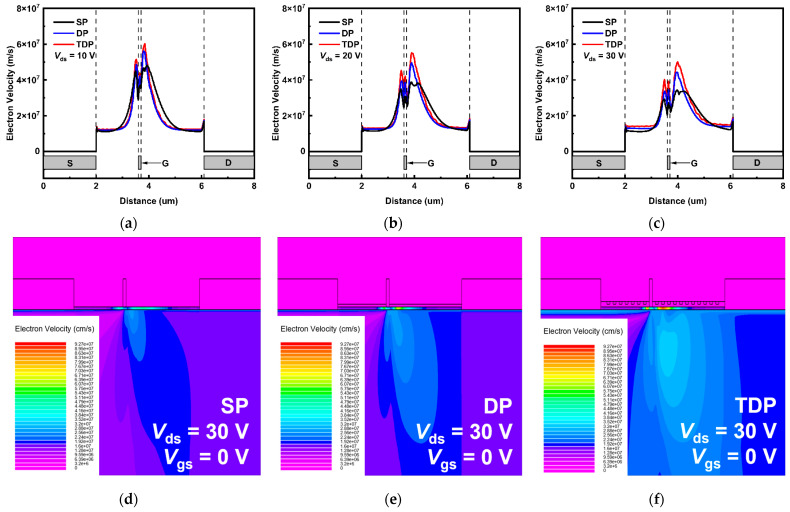
Electron drift velocity of junctional region in the SP, DP and TDP AlGaN/GaN HEMTs biased at (**a**) *V*_ds_/*V*_gs_ = 10 V/0 V, (**b**) *V*_ds_/*V*_gs_ = 20 V/0 V and (**c**) *V*_ds_/*V*_gs_ = 30 V/0 V. For *V*_ds_ = 30 V, cross-sectional electron velocity distributions of the (**d**) SP, (**e**) DP, and (**f**) TDP AlGaN/GaN HEMTs are presented.

In Figure 8a–c, the electron drift velocity values along the channel of the devices biased at *V*_ds_/*V*_gs_ = 10 V/0 V, 20 V/0 V and 30 V/0 V are presented. The relationship between electron drift velocity and electron mobility can be described in terms of the influence of the electric field. Specifically, electron drift velocity refers to the average speed at which electrons move under the influence of an electric field, while electron mobility quantifies the drift velocity of electrons per unit electric field strength. This relationship can be mathematically expressed as follows [29]:

(4) $$v_{d}=\mu E$$

where *v_d_* is electron drift velocity, *µ* is electron mobility and *E* is electric field. The electric field increases with higher *V*_ds_. Under the same *V*_ds_ condition (i.e., the same *E*), *v_d_* is directly related to *µ.* Due to varying degrees of self-heating effects, the TDP device exhibits the highest *µ* compared to DP and SP devices. Consequently, it also possesses the highest *v_d_* along the channel. In low electric field conditions, this relationship remains linear, with *v_d_* increasing proportionally to the *E*. However, at high electric field intensities, the *v_d_* may approach saturation due to increased scattering events between electrons and the lattice, limiting the maximum achievable *v_d_*. As a result, with *V_ds_* increasing from 10 V to 30 V, all devices present the *v_d_* decrease. However, for any given *V_ds_*, the *v_d_* of the TDP device is always higher than that of the DP and SP devices, as shown in Figure 8d,e.

## 4. Conclusions

In this paper, AlGaN/GaN HEMTs with TDP layers are systematically studied using the TCAD Silvaco simulation tool. Compared to conventional SP devices and DP devices, TDP devices exhibit superior thermoelectric characteristics in mitigating self-heating effects. The peak junction temperature of TDP devices is 386.36 K biased at *V*_ds_/*V*_gs_ = 30 V/0 V, which is much lower than DP (404.58 K) and SP (447.59 K) devices. The simulations show that TDP devices exhibit higher electron mobility and drift velocity, which lead to a higher *I*_dss_ of 1.266 A/mm, higher *G*_mmax_ of 0.329 S/mm and lower *R*_on_ of 2.64 Ω·mm than DP and SP devices because of better heat-dissipation capability. The TDP devices resolve the traditional trade-off between thermal dissipation and high-frequency operation in GaN HEMTs, making it particularly suitable for high-power-density and high-frequency applications. This capability holds great potential for enhancing the stability and reliability of the devices.

## Figures and Tables

**Table 1:** Calibration semiconductor parameters for GaN and AlGaN.

Parameters	GaN	AlGaN
Eg300 (eV)	3.4	3.96
Affinity (eV)	-	3.82
Align	0.8	0.8
Permittivity	9.5	9.5
Mun (cm^2^/V-s)	900	600
Mup (cm^2^/V-s)	10	10
Vsatn (cm/s)	2 × 10^7^	-
Nc300 (/cm^3^)	1.07 × 10^18^	2.07 × 10^18^
Nv300 (/cm^3^)	1.16 × 10^18^	1.16 × 10^18^

## Data Availability

The data that support the findings of this study are available from the corresponding authors upon reasonable request.
